# Balloon sinuplasty, an initial assessment: 10 cases, results and follow-up

**DOI:** 10.1590/S1808-86942010000500009

**Published:** 2015-10-22

**Authors:** João Flávio Nogueira Júnior, Aldo Cassol Stamm, Shiley Pignatari

**Affiliations:** 1 ENT Physician; 2 PhD in Otorhinolaryngology, Director of the Otolaryngology and Speech and Hearing Center of São Paulo - Hospital Prof. Edmundo Vasconcelos; 3 PhD in Otorhinolaryngology, Head of the Pediatric Otolaryngology Department of the Otolaryngology and Speech and Hearing Center of São Paulo - Hospital Prof. Edmundo Vasconcelos. Centro de Otorrinolaringologia e Fonoaudiologia de São Paulo - Hospital Professor Edmundo Vasconcelos

**Keywords:** video-assisted surgery, endoscopy, surgical procedures, minor.

## Abstract

**Abstract:**

Introduction and aims: Balloon sinuplasty has been the object of recent discussions and papers. It is believed that the use of these tools can bring benefits, when compared with traditional endoscopic sinus surgery. Although there already are papers on the efficacy of this new instrument in the literature, there is no study in our country with a series of cases and follow-up of patients undergoing sinuplasty. Our study aims to review the information of 10 patients who underwent balloon sinuplasty, alone or in combination, discussing the indications, complementary therapy and follow up evaluation.

**Method:**

retrospective study.

**Results:**

Of 10 patients, 6 were males and 4 females. Their ages ranged from 7 to 58 years. All patients had chronic rhinosinusitis without nasal polyps, of which 8 are associated with allergic disease. 3 patients underwent sinuplasty only, and 7 had other procedures done during the same procedure. Follow-up ranged from 2 to 7 months. Of the 10 patients, 9 showed symptoms improvement in imaging studies.

**Conclusion:**

sinuplasty was successfully performed in all patients, without major technical difficulties or complications. This instrument can become an alternative surgical treatment for some groups of patients.

## INTRODUCTION

Endonasal endoscopic surgery (EES) is the current method of choice for the surgical treatment of patients with nasosinusal inflammatory diseases who have clinical complaints and changed exams even after maximum clinical treatment^1^, ^2^, ^3^.

The goal of EES is to increase ventilation and drainage of the paranasal sinuses (PNS) involved, enabling the proper functioning of mucociliary movements in the nasal and PNS mucosa, thus facilitating the drainage of these cavities and enabling the penetration of medication and solutions for nasal flushing^1^, ^2^, ^3^, ^4^.

This method, despite bearing numerous benefits when compared to the conventional open procedures, still has some inherent challenges and limitations, especially because it ends up removing bone tissue and nasal mucosa fragments, which can cause bleeding, temporary physiological changes to the nasosinusal mucosa, especially the paradox reduction of mucociliary movements in the post-operative period and local scar fibrosis, which can cause re-obstruction of the treated PNS^1^,^2^.

The recent finding of high levels of nitric oxide (NO) in the nose and PNS in healthy persons in comparison with the tracheobronchial tree is also a current object of study; nonetheless, very little is known about the role of this gas in the nose and PNS, and which are the clinical and surgical implications of this substance in the upper airways. What is known is that the larger openings of the PNS ostia can significantly reduce the concentration of this gas inside these cavities^5^, ^6^, ^7^.

Considering these and other complications, many are the patients who currently undergo EES, including children, whom could benefit from potentially less invasive methods than EES.

As of 2006, a new device gained the lights in our specialty: the possibility of doing a balloon dilatation of the PNS ostia. This device came up with great fuzz in the USA for the enlargement of PNS ostia, especially the maxillary, frontal and sphenoidal sinuses for the treatment of nasosinusal inflammatory diseases^1^,^3^,^4^,^8^.

This device dilates PNS ostia and its adjacent structures, causing local micro-fractures, through the use of a balloon capable of withstanding high pressures. It is believed that after this dilation with the micro-fractures there is a remodeling of the treated PNS system drainage^1^,^8^,^9^.

In some recently published papers, it is believed that these devices may cause benefits, when compared to conventional EES, in reducing surgery time, hospital stay and the use of other materials, such as nasal packing^1^,^4^,^10^.

Although we already have papers in the world literature on the efficacy of this new device, with patient follow up of up to 2 years^11^, in our country there is no study of any series and follow up of patients submitted to sinuplasty.

## OBJECTIVES

Our goals were:

a) To review the information of 10 patients who were submitted to surgery for the treatment of inflammatory nasosinusal diseases in which this approach was used alone or in combination with other techniques: PNS balloon dilatation.
b) To discuss the indications and complementary treatment of these patients.
c) To assess the post-operative follow up of these patients.

## MATERIALS AND METHODS

After approval from the Ethics Committee of the Institution (021/2009), we carried out a retrospective study assessing the information present in the clinical, surgical and anesthetic charts and complementary exams of the 10 patients, all submitted to surgical treatment for nasosinusal inflammatory diseases.

**Figure 1:**
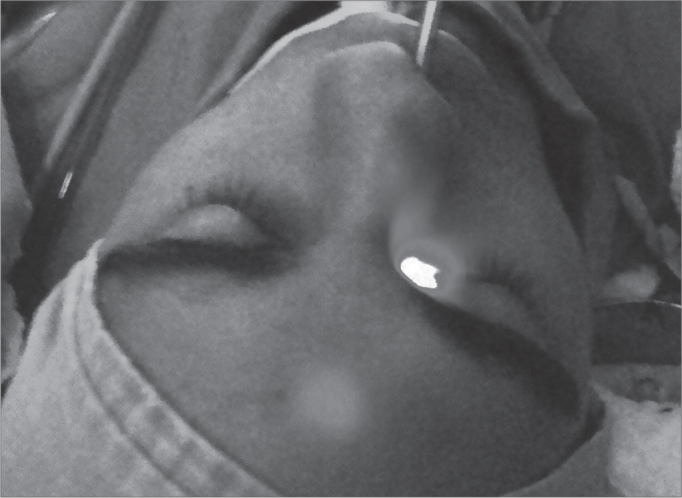
Right frontal sinus catheterization by means of transillumination. Notice a shining point in the patient's frontal region. With this image at hand, one can be sure of frontal sinus catheterization.

**Figure 2:**
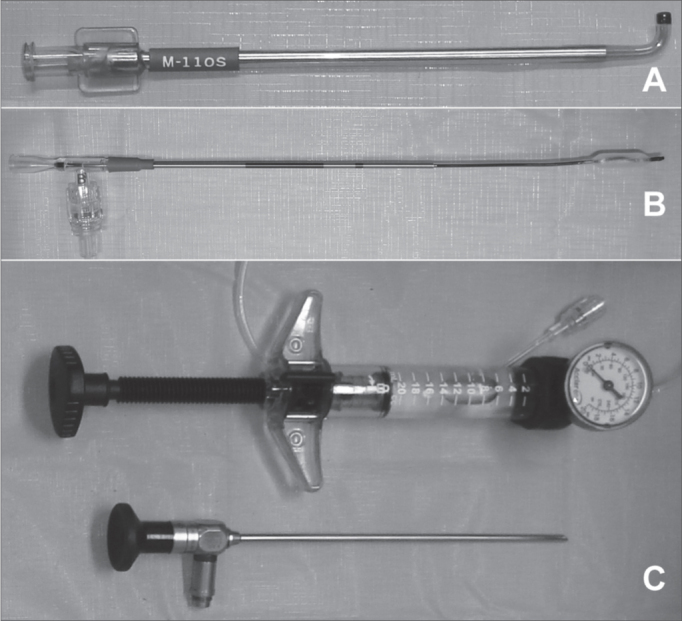
Material used in the sinuplasty. A: Guide catheter. There are different types of catheters with different angles, according with the paranasal sinus to be catheterized. In this case we show the catheter to be used in the maxillary sinus of the pediatric patient. B: 5mm balloon used. C: Pump used to inflate the balloon at high pressure and angled scope (45°. and 4 mm in diameter).

The inclusion criteria for these patients was the use alone or combined of some other surgical procedure involving balloons for the dilation of the natural drainage ostia of the PNS, the sinuplasty carried out under transillumination (Fig. 1) with the Relieva Sinus Balloon Catheter System (Acclarent, INC, Menlo Park, California), made up by guide catheters, illumination catheter for PNS catheterization and balloons of 5mm in diameter (Fig. 2). The exclusion criterion was not using these balloons.

We analyzed patient data such as demographics, surgery indication, prior surgeries, paranasal sinuses treated during the procedure, other procedures carried out in the same surgery, use of nasal packing and other materials, surgery duration, hospital stay duration, technical difficulties, possible complications and post-op follow up.

## RESULTS

All the patients were submitted to surgery in the same institutions, done by three different surgeons from March through August of 2009. All the procedures were done under general anesthesia (Table 1).

**Table 1:** Patients enrolled.

Gender	Age	Clinical complaint	Treated PNS / Procedures	Follow up
M	52	Chronic rhinosinusitis (CRS) with frontal headache	Frontal bilateral (revision case)	7 months. Complaints after surgery and frontal abscess 3 months after sinuplasty. Draf III procedure.
M	7	CRS with nasal obstruction	Left Maxillary	7 months without clinical complaints, CT scan improvement
M	21	CRS with frontal headache and nasal secretion	Right frontal + septoplasty	7 months without clinical complaints, CT scan improvement
F	32	CRS with frontal headache (revision), nasal secretion and orbital pain	l Left frontal (revision)	6 months without clinical complaints, CT scan improvement
F	17	CRS with facial pain and nasal secretion	Right Maxillary + septoplasty	6 months without clinical complaints, CT scan improvement
F	58	CRS with facial pain, nasal secretion, after dental implant.	Left maxillary + septoplasty + left-side maxillary antrostomy	5 months without clinical complaints, CT scan improvement
F	30	CRS with headache and facial pain	Maxillary bilateral + septoplasty	5 months without clinical complaints, CT scan improvement
M	25	CRS with frontal headache	Frontal bilaterally + septoplasty	4 months without clinical complaints and CT scan improvement
M	18	CRS. Patient with brain palsy.	Right maxillary and frontal + septoplasty + medial partial turbinectomy	3 months with clinical and CT scan improvements
M	26	CRS with frontal headache and nasal secretion	Frontal bilaterally + septoplasty	2 months without clinical complaints and CT scan improvements

Demographics:

Of the 10 patients, 6 (60%) were males and 4 (40%) were females. Their ages varied between 7 and 58 years, mean of 28.6 years.

Indications:

The indication for using balloons, after discussions with the patient or Family members was left in the hands of the surgeons who performed the procedures; however, all the patients had chronic rhinosinusitis (CRS) and no nasal polyps; 8 (80%) of them had nasosinusal disease associated.

The most common symptom found was frontal headache, followed by nasal secretion with postnasal drip and facial pain.

Previous nasosinusal surgeries:

Of the 10 patients, 8 (80%) had never been operated to treat inflammatory nasosinusal diseases. In two cases (20%) the dilations were carried out through EES, and they had recurrence of their clinical complaints and of the alterations in complementary exams (nasofibroscopy and CT scan). These two patients had allergic characteristics.

Treated paranasal sinuses:

In the 10 patients, 13 PNS were catheterized and treated with sinuplasty: 8 frontal sinuses (3 bilateral, 1 on the right side and 1 on the left side) and 5 maxillary sinuses (1 bilateral, 2 on the right side and 1 on the left side). Of these, even after balloon dilatation, 2 PNS were open in the traditional way: 1 maxillary sinus (left) in the same surgery and the frontal sinus of another patient - but this one in another surgery in which the Draf III procedure was carried out.

Surgical procedures carried out:

In 3 patients (30%), sinuplasty was carried out alone and in 7 patients (70%) one septoplasty (conventional or endoscopic) was carried out in the same surgery. All the patients who had undergone septoplasty had never been submitted to any nasosinusal surgical treatment.

Of the patients who had undergone septoplasty, 1 underwent partial middle turbinectomy to treat bullous middle turbinate. No patient was submitted to inferior turbinectomy.

Nasal packing and other materials:

Of the 10 patients, 2 (20%) had Merocel® nasal packing in the middle meatus region. These two patients were submitted to traditional PSN opening after sinuplasty.

One patient had Merocel® inferior nasal packing. In the seven patients in whom septoplasty was carried out, one nasal “splint” was placed and fixed at the end of the procedure, and it was removed at the return visit.

Surgery time:

In order to evaluate the surgery time, we reviewed the anesthesia notes of the 10 patients. We did not take into account only the time it took for the procedure to be made, but also the beginning and end of the surgery, according to the form filled out by the anesthesiologist. This time varied between 40 and 120 minutes, mean time of 70 minutes per patient.

Hospital stay period:

Of the 10 patients, 7 were discharged on the day following surgery. Three patients we discharged on the very day of the procedure. These three patients were the same ones submitted to sinuplasty only, without other simultaneous procedures.

Technical difficulties and complications:

All PNS previously defined for treatment were successfully catheterized and dilated. There were no major technical difficulties in doing the sinuplasty and there were no intraoperative complications. There was one immediate post-operative bleeding with the need for inferior nasal packing.

Postoperative follow up:

All the patients returned for a post-op visit and the follow up varied between 2 and 7 months. Of the 10 patients, 9 (90%) improved in their symptoms - according to answers from the patients, and had better image studies (CT scan). No patient had late post-operative bleeding, nonetheless, 7 patients had nasal obstruction on the first week of post-op. These were the patients who suffered concurrent septoplasty.

In two patients, more frequent nasal cleanings were carried out because of nasal crusts. These patients were submitted to traditional procedures, even after PNS dilation.

One patient had bilateral frontal sinus re-obstruction. This patient had already undergone prior EES.

## DISCUSSION

The use of these new devices in patients has already been proven safe, with good results, being specifically indicated for patients with CRS^1^,^3^,^4^,^8^,^11^,^12^. In our cases, all the patients had CRS, and 8 of them had allergic symptoms associated. Of these, 7 were adults and 1 was a child (7 years of age). All the 7 adults had already been submitted to clinical treatment with partial improvement in their image exams (CT scan); however, without relevant improvements in symptoms. The child had undergone adenoidectomy 2 years before, and had also been clinically treated, without improvements in her chronic sinus disorder.

**Figure 3:**
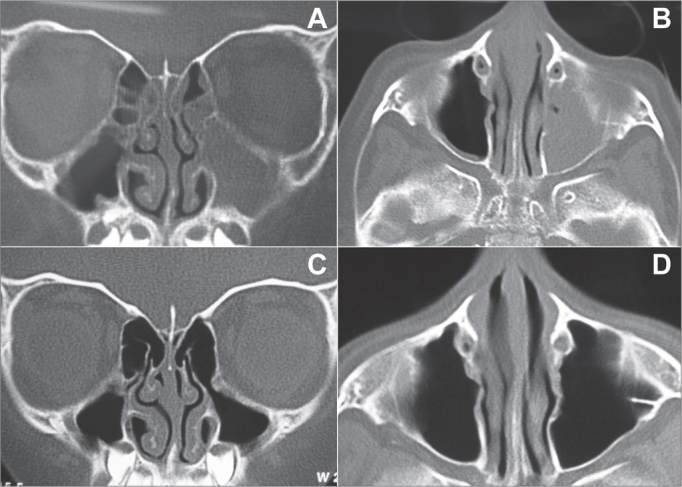
Pre and post-operative CT scan (exam carried out 6 months after the surgery) from a patient submitted to sinuplasty. A: Coronal section of a pre-operative image showing complete blurring of the left maxillary sinus and some ipsilateral ethmoidal cells. B: axial section of a pre-operative image showing blurring of the left maxillary sinus. C: post-operative image (6 months after the procedure) in a coronal cross-section showing normal aeration of the left maxillary sinus and ipsilateral ethmoidal cells. Notice the dilatation seen in the region of the maxillary infundibulum. D: Post-operative image (6 months after the procedure) in an axial cross-section showing normal aeration of the left maxillary sinus.

One of the most promising indications for sinuplasty is patients with CRS. Doing EES in children with CRS is not common and is controversial as far as its indication and efficacy are concerned, hence the difficulty of having a series of cases with a large number of pediatric patients. Nonetheless, in a recent paper, 30 children were submitted to sinuplasty. No complication was seen and good postoperative results were obtained from this less invasive technique^13^. In our pediatric patient we also noticed an important improvement in symptoms and in the complementary image exams (Figure 3).

All the patients, even those who had already been submitted to EES, were treated with specific medication in the pre-operative period, for the time advocated in the literature.

Numerous papers illustrate the fact that patients with allergies tend to have more complaints of recurrent symptoms, both with clinical treatment and even after EES^1^,^14^. Of the 10 patients, the 8 with allergic characteristics submitted to pre-operative clinical treatment also presented recurrent symptoms and image exams (CT scan) with suggestive signs of mucous edema or PNS blurring; nonetheless, even with the relatively short follow up we have, only one patient had recurrent complaints after sinuplasty, with alterations of the postoperative image exam (CT scan), showing local restenosis in the region of the frontal sinus recess, bilaterally.

This patient did only sinuplasty, without other procedures associated. Nonetheless, this same patient had already been submitted to numerous previous surgeries to treat frontal CRS, always having local re-obstruction and recurrent symptoms. It may be that the failure of the sinuplasty is more associated with a possible incorrect and inaccurate indication than the technique and devices used. Three months after doing the sinuplasty, the patient had to be submitted to traditional EES (Draf III) to drain an abscess in the frontal sinus region.

The other patient who underwent revision surgery using only sinuplasty in a region previously altered by EES, so far has improved on his symptoms and in the image exams (CT Scan), without alterations, even being the bearer of allergic characteristics.

It is important to mention that in these allergic patients, even after doing sinuplasty, clinical treatment continues to be done with specific medication.

**Figure 4:**
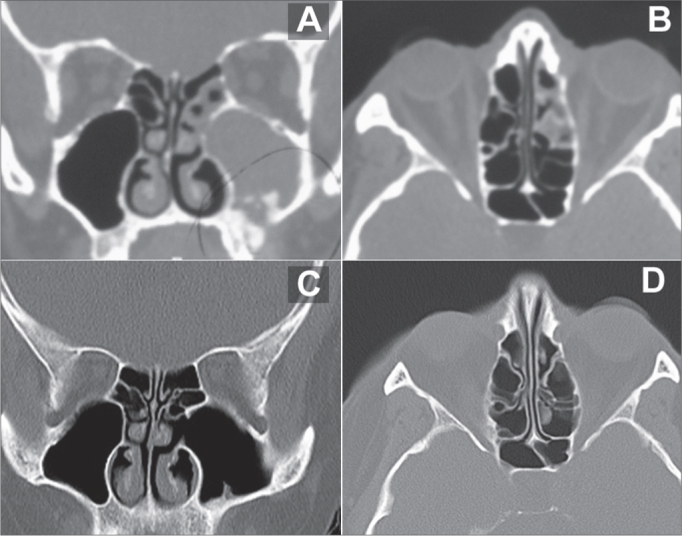
Pre and post-op CT scan of the patient submitted to septoplasty, left maxillary sinuplasty and conventional maxillary antrostomy on the left side. A: Coronal section of a pre-operative exam showing a lesion on the floor of the left maxillary sinus, images with bone density inside the cavity, complete blurring of the cavity and of the ipsilateral anterior ethmoidal cells. The bone density images correspond to bone fragments from the maxillary sinus floor elevation done in order to harbor dental implants. B: Axial cross-section of a pre-operative image showing partial blurring of the anterior ethmoidal cells on the left side. Notice that the limit is exactly on the basal lamella of the middle concha, which separates the anterior ethmoidal cells from the posterior ones. C: Postoperative exam (5 months after the procedure) in a coronal cross-section showing the left-side maxillary antrostomy and normal aeration of the maxillary cavity and ethmoidal cells. D: Post-operative exam (5 months after the procedure) in an axial cross-section showing normal aeration of ethmoidal cells.

Among the non-allergic patients submitted to sinuplasty, 1 had had chronic sinus disease for 6 months, after a dental implant - This patient had, upon CT scan, small bone-density images inside his right maxillary sinus and complete blurring of the same PNS and in his ipsilateral anterior ethmoidal region. These small images corresponded to fragments of the bone graft done by the dentist in order to elevate the maxillary sinus floor to anchor the dental implants (Figure 4).

In this patient, even after sinuplasty and exhaustive flushing of the maxillary cavity, with abundant oozing, we did a broad maxillary antrostomy, since the surgeon needed to be sure of the complete removal of all the bone fragments. After the broad antrostomy and visualization of the maxillary cavity with an angled endoscope, there was no need for further flushing to remove eventual residual bone fragments.

**Figure 5:**
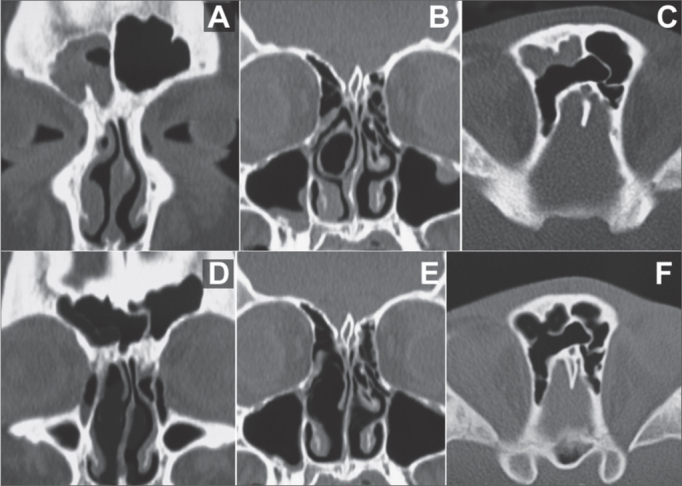
Pre and post-operative CT scan of a patient submitted to septoplasty, partial middle turbinectomy, right-side maxillary and frontal sinuplasty and conventional frontal sinusectomy on the right side. A: coronal cross-section pre-operative image showing right-side frontal sinus blurring. B: Coronal cross-section pre-operative image showing septal deviation, bullous right-side middle turbinate and images showing soft tissue on the floor of the right maxillary sinus. C: Axial, pre-operative view showing right frontal sinus blurring. Notice the presence of supraorbital ethmoidal cell, representing a challenge in the proper identification of the true frontal cavity. D: Post-operative exam (3 months after the procedure) in a coronal cross-section showing normal aeration of the right-side frontal cavity. E: Post-operative coronal cross-section showing the results from the partial right-side middle turbinectomy and the septoplasty. F: Axial cross-section of the post-operative exam showing normal aeration of the right-side frontal sinus. Notice the shunt created with the supraorbital ethmoidal cell through conventional frontal sinusectomy.

In another patient without allergic disease, there was an important anatomical alteration (bullous middle turbinate) which would be a potential cause of right-side middle meatus obstruction and, consequently, obstruction to the drainage of the right-side frontal and maxillary sinuses. This patient was submitted to partial middle turbinectomy with removal of the meatal pars of the right-side middle turbinate and later right-side maxillary and frontal sinuplasty (Figure 5). It may be that most of the nasosinusal problemsof this patient are due to meatal obstruction caused by the bullous component of the middle turbinate, and sinuplasty was indicated in this patient because he had brain palsy, and this could hamper dressings and postoperative care in cases of conventional EES.

Sinuplasty may be very interesting in patients who have problems concerning postoperative dressings, such as children and those with some type of mental impairment^13^,^15^,^16^.

Not removing tissue from the nose and paranasal sinuses can represent a major advantage, having seen that problems such as transitional postoperative changes in the nose and PNS mucociliary movements, scar fibrosis and subsequente local re-stenosis, synechia and postoperative bleeding are much less frequent in patients submitted to these dilatations, according to papers recently published^1^,^4^,^11^,^15^, ^16^, ^17^.

**Figure 6:**
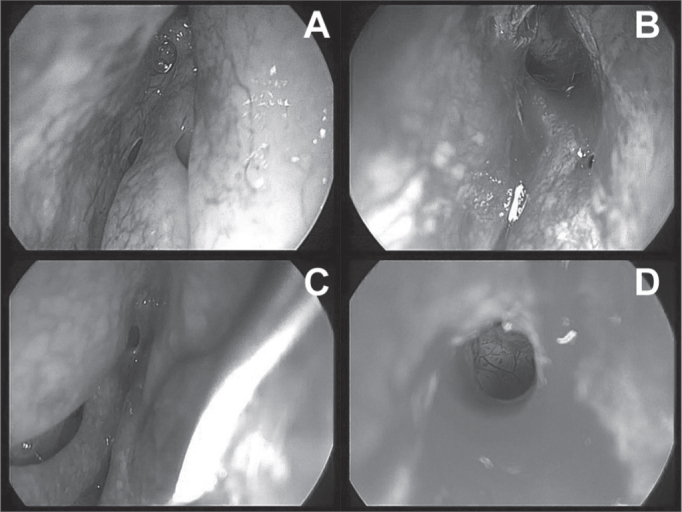
Endoscopic image of the sinuplasty. A: image from the 45° and 4 mm diameter scope from the left frontal sinus recess before the balloon dilatation. B: endoscopic image with the same angled device showing dilatation and visualization of inside the left frontal sinus. Notice that there was no removal of mucous or bone tissue and even then we had a satisfactory view. C: endoscopic image with the same angled device from the right frontal sinus recess region. D: endoscopic image after dilatation.

We noticed this in those patients who underwent sinuplasty without other associated procedures. In them, sinuplasty alone enabled opening the PNS drainage (Figure 6), postoperative recovery was much faster and with less need to clean the nose. Notwithstanding, patients who had undergone concurrent sinuplasty and septoplasty in the morning, had important nasal bleeding in the afternoon, when already in their rooms. One inferior nasal packing was carried out, and it was removed on the following morning, without associated bleedings.

All the patients were submitted to the procedures under general anesthesia, although recent publications discuss the possibility of doing sinuplasty procedures safely under local anesthesia in an outpatient basis, with or without sedation^11^,^12^.

As to surgery duration, it is important to stress that it is not only the surgical procedure time, but also the entire anesthesia time, taken from the charts which were filled out by the anesthesiologists in the operating rooms. We did not carry out any comparative study as to surgery duration in those patients who underwent conventional EES versus sinuplasty. However, we can say that the surgery time was shorter in the three patients who were submitted to sinuplasty alone, when compared to those whom, besides sinuplasty were also submitted to other concurrent surgical interventions, such as septoplasty and middle turbinectomy. Even then, the maximum time duration of the anesthesia for these procedures was of 120 minutes, which is similar to EES duration- in accordance with the literature^10^. We must also stress that the first procedures carried out took longer, and this is due to the learning curve concerning the use of these new devices and the time it takes to set up the equipment necessary to do the EES and the sinuplasty.

Although in the 10 patients of this series we were able to successfully catheterize and dilate all the PNS attempted, there were patients outside of this series in whom we were unable to catheterize the left frontal sinus because of anatomical and technical difficulties pertaining to the surgeon and the patient. In this case, a conventional EES was carried out. Some authors have also faced a similar problem, especially in cases of nasosinusal hypoplasia^13^.

As to the reduced number of patients included, it is important to stress that this is a pioneering study in our country, with a device that is relatively new in the world and it was only approved in Brazil in 2009. There are similar reports in the literature, with an even lower number of patients^15^, having seen that the set of balloons have a relatively high cost, although it is already available from some health insurance companies.

Each patient used a different set of dilatation balloons; however, when necessary, in the same patient, the same balloon was used in different PNSs. Although there are comparative cost analyses in the literature showing that the cost of sinuplasty is similar to that of conventional EES in the United States^10^, we did not perform cost comparisons with patients who underwent EES alone. Nonetheless, among the patients submitted to sinuplasty alone, no other special material, such as nasal packing and nasal “splint” was utilized. We believe that the cost of these other materials is not an important factor in the total cost of EES, while the cost of the sinuplasty equipment can be relevant considering the total expenditures of the patients.

There is a general consensus that the surgeries are indicated when there is maximal clinical treatment failure, or in special cases, with associated anatomical alterations. Surgery is carried out so that we can open the PNS ostia to promote ventilation, proper secretion drainage and penetration of topical medication^1^,^18^. Notwithstanding, a frequent doubt surgeons have is the size of PNS ostia broadening.

There is no consensus on the size or shape of the PNS openings; nonetheless, we know that the ventilation and the use of topical medication are better with broader openings.

In a recent publication, a group of researchers tried to identify the minimum size of the maxillary antrostomy so that both ventilation and topical medication were efficient. This size was of 3.96 mm in diameter^18^. We used 5 mm balloons, but we cannot be sure that after dilatation, these regions remain with such diameter.

In a recent publication, a group of physicians assessed the long term results of the bullous middle turbinate treatment by only crushing the bullous component, without tissue removal. In most of the patients assessed who used this technique, the bullous component of the turbinate recurred. Although there is no evidence for such, the authors extrapolated the result for possible implications such as sinuplasty, theorizing that simple dilatation or crushing structures such as the agger-nasi could represent a temporary solution^19^.

As far as symptoms are concerned, we did not use specific evaluation questionnaires concerning quality of life or symptoms. In our study, symptoms improving or worsening were based on the notes from the charts of the 10 patients who answered the questions of the physicians in outpatient visits.

As to the patients who underwent other procedures in the same sinuplasty, 7 underwent septoplasty and one of them also underwent medium partial turbinectomy. These other procedures were previously indicated by the surgeons to correct septal deviations seen upon CT scans and also to help visualize the middle meatus region. All these patients had not undergone previous surgeries. On the 3 patients who underwent only sinuplasty, but not septoplasty, 2 had already been submitted to previous septoplasty and 1, despite a septal deviation, it was a 7-year-old child, did not undergo septoplasty.

## CONCLUSIONS

In these 10 patients who were submitted to sinuplasty alone or in combination with other techniques, after 7 months, 9 (90%) improved in their symptoms and in their exam images. The most frequent indication to use this new device was in patients with CRS, with or without allergies, not responsive to maximum clinical treatment.

Sinuplasty was successfully carried out in all the patients, without major technical difficulties or intraoperative complications. We also did not notice post-operative complications. Nonetheless, the comparative analysis of the surgical time and costs was not carried out. This device can become an interesting alternative to the surgical treatment of some groups of patients, since it introduces a minimally invasive procedure which enables nasal and paranasal sinuses mucosa preservation. Nonetheless, prospective studies and case series studies are required, with a larger number of patients and longer follow up is necessary for a better understanding of the role of sinuplasty in the treatment of paranasal sinuses inflammatory diseases.

## References

[1] Nogueira Júnior JF, Silva MLS, Santos FP, Stamm AC (2008). Sinuplastia com balão: um novo conceito na cirurgia endoscópica nasal. Arq Int Otorrinolaringol..

[2] Stamm A, Nogueira JF, Lyra M (2009). Feasibility of balloon dilatation in endoscopic sinus surgery simulator. Otolaryngol Head Neck Surg..

[3] Friedman M, Schalch P (2006). Functional endoscopic dilatation of the sinuses (FEDS): patient selection and surgical technique. Op Tech Otolaryngol Head Neck Surg..

[4] Bolger WE, Brown CL, Church CA, Goldberg AN, Karanfilov B, Kuhn FA (2007). Safety and outcomes of balloon catheter sinusotomy: a multicenter 24-week analysis in 115 patients. Otolaryngol Head Neck Surg..

[5] Andersson JA, Cervin A, Lindberg S, Uddman R, Cardell LO (2002). The paranasal sinuses as reservoirs for nitric oxide. Acta Otolaryngol..

[6] Djupesland PG, Chatkin JM, Qian W, Haight JS (2001). Nitric oxide in the nasal airway: a new dimension in otorhinolaryngology. Am J Otolaryngol..

[7] Kirihene RK, Rees G, Wormald PJ (2002). The influence of the size of the maxillary sinus ostium on the nasal and sinus nitric oxide levels. Am J Rhinol..

[8] Brown CL, Bolger WC (2006). Safety and feasibility of balloon catheter dilation of paranasal sinus ostia: a preliminary investigation. Ann Otol Rhinol Laryngol..

[9] Bolger WE, Vaughan WC (2006). Catheter-based dilation of the sinus ostia: initial safety and feasibility analysis in a cadaver model. Am J Rhinol..

[10] Friedman M, Schalch P, Lin HC, Mazloom N, Neidich M, Joseph NJ (2008). Funcional endoscopic dilation of the sinuses: patient satisfaction, postoperative pain, and cost. Am J Rhinol..

[11] Levine HL, Sertich AP 2nd, Hoisington DR, Weiss RL, Pritikin J (2008). Multicenter registry of balloon catheter sinusotomy outcomes for 1,036 patients. Ann Otol Rhinol Laryngol..

[12] Vaughan W (2008). Review of balloon sinuplasty. Curr Opin Otolaryngol Head Neck Surg..

[13] Ramadan HH (2009). Safety and feasibility of balloon sinuplasty for treatment of chronic rhinosinusitis in children. Ann Otol Rhinol Laryngol..

[14] Nayak DR, Balakrishnan R, Murty KD (2001). Endoscopic physiologic approach to allergy-associated chronic rhinosinusitis: a preliminary study. Ear Nose Throat J..

[15] Wittkopf ML, Becker SS, Duncavage JA, Russell PT (2009). Balloon sinuplasty for the surgical management of immunocompromised and critically ill patients with acute rhinosinusitis. Otolaryngol Head Neck Surg..

[16] Slow JK, Al Kadah B, Werner JA (2008). Balloon sinuplasty: a current hot topic in rhinology. Eur Arch Otorhinolaryngol..

[17] Brehmer D (2008). Catheter-based balloon dilatation of the frontal, maxillary, and sphenoid ostia: a new procedure in sinus surgery. HNO..

[18] Grobler A, Weitzel EK, Buele A, Jardeleza C, Cheong YC, Field J (2008). Pre- and postoperative sinus penetration of nasal irrigation. Laryngoscope..

[19] Kieff DA, Busaba NY (2009). Reformation of concha bullosa following treatment by crushing surgical technique: Implication for balloon sinuplasty. Laryngoscope..

